# Green Catalysis in Nanomaterials—Photocatalysis and Electrocatalysis

**DOI:** 10.3390/nano15010005

**Published:** 2024-12-25

**Authors:** Longlu Wang, Yongqi Liu, Chengbin Liu

**Affiliations:** 1 College of Electronic and Optical Engineering & College of Flexible Electronics (Future Technology), Nanjing University of Posts and Telecommunications, Nanjing 210023, China; 1223025001@njupt.edu.cn; 2 College of Chemistry and Chemical Engineering, Hunan University, Changsha 410082, China

## 1. Introduction

At present, the world is facing urgent challenges of energy shortages and environmental pollution, which drives the need for green and sustainable solutions [1,2]. To address the escalating energy crisis and reduce reliance on depleting fossil fuels, various strategies for building sustainable energy systems have been proposed. Among these, green catalysis via photocatalytic and electrocatalytic processes has become a promising approach, especially in the field of nanomaterials [3,4,5]. These catalytic systems use solar energy and electrical input to drive key reactions such as hydrogen production, pollutant degradation, and energy conversion, promoting the development of renewable and environmentally friendly energy while offering solutions to energy shortages and environmental pollution [6,7].

Photocatalytic and electrocatalytic technologies have broad applications and tremendous development potential in the field of green catalysis [8,9,10]. Photocatalysis uses solar energy to drive chemical reactions based on the band structure of semiconductor materials. It excites semiconductor catalysts by light irradiation, converting light energy into chemical energy with significant environmental benefits [11,12]. Electrocatalysis accelerates chemical processes through electrochemical reactions by using catalysts on electrodes to lower the activation energy of reactions, thereby increasing efficiency [13]. This allows for more efficient energy conversion. Nanomaterials offer significant advantages in photocatalytic and electrocatalytic reactions due to their unique physical and chemical properties (such as high specific surface area and excellent electron transport capabilities). The use of nanomaterial catalysts enables photocatalytic and electrocatalytic processes to become more controllable and efficient, thus achieving low-pollution green catalysis [14,15].

Improving the efficiency of photocatalytic and electrocatalytic processes depends critically on the careful design and optimization of nanomaterials [16]. In addition, the selection of cost-effective and environmentally friendly materials can further advance the realization of green catalysis, providing scalable solutions for energy conversion and environmental remediation, placing nanomaterial-based green catalysis technologies at the forefront of sustainable energy applications.

## 2. An Overview of Published Articles

Yin et al. (Contribution 1) reviewed recent advances in the study of metal centers and their coordination environments in carbon-supported single-atom catalysts (C-SACs) during the oxygen reduction reaction (ORR) via the 2e^−^ pathway (2e^−^-ORR). First, they discussed the reaction mechanism of 2e^−^-ORR at the active sites of C-SACs. Then, they summarized the structural regulation strategies for 2e^−^-ORR active sites, including the metal active centers, types and configurations of nitrogen or heteroatom coordination, and nearby functional groups or substituents, offering new ideas for developing superior acidic 2e^−^-ORR electrocatalysts in C-SACs. Finally, they presented the current challenges and future opportunities for the acidic 2e^−^-ORR pathway on C-SACs, which will drive the development of distributed H_2_O_2_ electrosynthesis processes.

He et al. (Contribution 2) reviewed the latest research advances in MoS_2_-catalyzed hydrogen production. They explained the mechanisms by which MoS_2_ enhances catalytic performance, focusing on edge sites, sulfur vacancies, doping, and phases. They also conducted a deeper investigation into the catalytic mechanisms of MoS_2_. In addition, they proposed strategies to improve the hydrogen evolution performance of MoS_2_ based on its catalytic mechanism, such as combining first-principles calculations with in situ characterization techniques to explore its catalytic hydrogen evolution mechanism, and standardization by constructing micro/nanostructured devices to attribute the performance to catalytic active sites with specific atomic structures.

Liu et al. (Contribution 3) synthesized and reported a novel bifunctional electrocatalyst, C@CoP-layered nanosheets, composed of vertical CoP and a carbon framework induced by ZIF-67. The synthesized binder-free bifunctional electrocatalyst exhibited excellent performance in both the hydrogen evolution reaction and oxygen evolution reaction. They confirmed that the exceptional catalytic performance was attributed to the vertically layered nanosheet array structure. The introduction of the carbon framework effectively enhanced the conductivity, facilitated the electrolyte transport, and increased the exposure of the active sites.

Alzarea et al. (Contribution 4) prepared well-dispersed Pd-Cu nanoparticles (NPs) on a Co-Cr layered double hydroxide (LDH) support by combining in situ co-precipitation/hydrothermal and sol immobilization techniques. In their study, the bimetallic Pd-Cu nanoparticles supported on Co-Cr LDH exhibited excellent performance in the aerobic oxidation of benzyl alcohol and the reduction of nitrobenzene. They attributed the excellent catalytic performance to the uniform distribution and small size of Pd-Cu NPs on the surface of Co-Cr LDH, which act as active sites. In addition, the prepared catalyst showed excellent stability during repeated operation.

Ye et al. (Contribution 5) reviewed recent advancements in piezoelectric photocatalysis. They first introduced the mechanisms of photocatalysts and the principles of the piezoelectric effect, briefly explaining how the piezoelectric effect influences photocatalysis. Next, they provided a comprehensive overview of existing piezoelectric photocatalysts, followed by a discussion of their various application scenarios. Finally, the authors summarized the current challenges and future directions in the field. This review broadens research perspectives on piezoelectric photocatalysts and promotes their application across a wider range of fields.

## 3. Conclusions

In summary, the articles contributed to this Special Issue focus on presenting the state of the art of green catalysis technologies in nanomaterial-based photocatalysis and electrocatalysis. They provide detailed descriptions of the unique properties of nanomaterials, with an emphasis on their role in environmental protection, energy conversion, and enhancement in catalytic efficiency and selectivity.

In addition, they discuss how nanomaterials enhance light absorption in photocatalytic processes to drive chemical reactions, as well as their applications in electrocatalysis, particularly in energy conversion, which is a major focus of this topic. This Special Issue aims to summarize the current challenges of green catalysis in nanomaterial-based photocatalysis and electrocatalysis, propose future research directions, and promote the application of nanomaterials in these fields, especially in renewable energy and environmental technologies.
